# Binding of Human Fibrinogen to MRP Enhances *Streptococcus suis* Survival in Host Blood in a α_X_β_2_ Integrin-dependent Manner

**DOI:** 10.1038/srep26966

**Published:** 2016-05-27

**Authors:** Yaya Pian, Xueqin Li, Yuling Zheng, Xiaohong Wu, Yuan Yuan, Yongqiang Jiang

**Affiliations:** 1 State Key Laboratory of Pathogen and Biosecurity, Beijing Institute of Microbiology and Epidemiology, Beijing 100071, China; 2 Institute of Biophysics, Chinese Academy of Sciences, Beijing 100101,China

## Abstract

The Gram-positive bacterium *Streptococcus suis* serotype 2 (*S. suis* 2), an important zoonotic pathogen, induces strong systemic infections in humans; sepsis and meningitis are the most common clinical manifestations and are often accompanied by bacteremia. However, the mechanisms of *S. suis* 2 survival in human blood are not well understood. In our previous study, we identified muramidase-released protein (MRP), a novel human fibrinogen (hFg)-binding protein (FBP) in *S. suis* 2 that is an important epidemic infection marker with an unknown mechanism in pathogenesis. The present study demonstrates that the N-terminus of MRP (a.a. 283–721) binds to both the Aα and Bβ chains of the D fragment of hFg. Strikingly, the hFg-MRP interaction improved the survival of *S. suis* 2 in human blood and led to the aggregation and exhaustion of polymorphonuclear neutrophils (PMNs) via an α_X_β_2_ integrin-dependent mechanism. Other Fg-binding proteins, such as M1 (GAS) and FOG (GGS), also induced PMNs aggregation; however, the mechanisms of these FBP-hFg complexes in the evasion of PMN-mediated innate immunity remain unclear. MRP is conserved across highly virulent strains in Europe and Asia, and these data shed new light on the function of MRP in *S. suis* pathogenesis.

The Gram-positive bacterium *Streptococcus suis* serotype 2 (*S. suis* 2) is an emerging human pathogen that induces systemic infections in humans; sepsis and meningitis are the most common clinical manifestations and are often accompanied by bacteremia^1^,^2^,^3^. Severe sepsis from *S. suis* 2 leads to streptococcal toxic shock syndrome (STSS), which has a high incidence of morbidity and mortality despite antibiotic therapy^4^. To induce bacteremia, bacterial pathogens must survive in the blood and disseminate while evading polymorphonuclear leukocyte (PMN)-mediated phagocytosis. However, little is known about how this evasion is achieved by *S. suis* 2.

Fibrinogen (Fg) is a heterogeneous dimetric plasma glycoprotein consisting of three pairs of non-identical Aα, Bβ and γ peptide chains. Fg is the fundamental building block of insoluble fibrin clots and is well known for playing important roles in hemostatic processes^5^ and the innate immune system^6^. Numerous bacterial proteins that interact with Fg have been identified in Gram-positive species. Although many of these proteins employ distinct Fg-binding mechanisms^7^, they all manipulate the biology of Fg to enhance microbial survival in the host. Group A streptococci (GAS) avoid phagocytosis by M protein to inhibit complement activation^8^,^9^. FOG, a M1-like protein from group G streptococci, induce bacteria and PMN aggregation^10^. *Staphylococcus aureus* interfere with the recognition signal of phagocytes by Efb^11^. *Streptococcus agalactiae* form a semi-flexible polymer-like network by FbsA-Fg interaction, which becomes an efficient mask against phagocytic clearance^12^.

In silico analysis of the Chinese *S. suis* 2 reference strain 05ZYH33 genome revealed 33 putative cell wall-anchored proteins containing LPxTG or a related motif^13^; however, it remains to be determined whether these surface proteins play roles in *S. suis* pathogenesis; thus far, only an adhesin, FBP, was reported to bind to Fg^14^. In our recent study, a proteomics-based far-western blotting technique (PBFWB) was developed to identify the fibrinogen-binding surface proteins of *S. suis* (SsFBPs) on a large scale^15^. Muramidase-released protein (MRP) and Enolase are the major Fg-binding proteins of *S. suis* 05ZYH33, a Chinese pathogenic strain (NC_009442.1) isolated from a deceased STSS patient. *S. suis* Enolase functions as a protective antigen displayed on the bacterial cell surface^16^. However, Enolase is a functional important enzyme in both prokaryotic and eukaryotic organisms^17^ and its gene couldn’t be knocked out in our previous study^15^. The mechanism of Enolase in immune evasion remained unclear until now. MRP is a 136-kDa protein that associates with the cell wall of *S. suis* via an LPxTG motif but is also released into culture supernatants^18^; it is often reported as an epidemic infection marker for highly virulent strains in Europe and Asia and has low amino acid sequence similarity to other proteins with known functions^19^,^20^,^21^. In our previous study, rat antiserum to MRP had highly bactericidal activity against *S. suis* 05ZYH33 in human blood^22^, and the MRP-hFg interaction improved the antiphagocytic capacity of *S. suis* 2 against PMNs^15^, suggesting that MRP might be involved in the survival of the bacteria in the host blood. Therefore, the specific mechanism of MRP involvement in the evasion of innate immunity by *S. suis* in host blood needs to be clarified.

The results of this study indicate that MRP directly binds to the Aα and Bβ chains of the D fragment in hFg. Furthermore, the interaction of soluble (released) MRP with hFg enhanced the survival of *S. suis* 2 in human blood by disrupting the phagocytic activity of PMNs by inducing their aggregation in an α_X_β_2_ integrin-dependent manner. These results provide useful insight into the molecular mechanism of *S. suis* pathogenesis and the biological basis of MRP as an important infection marker.

## Results

### Localization of the hFg-binding region in MRP

**Figure 1:**
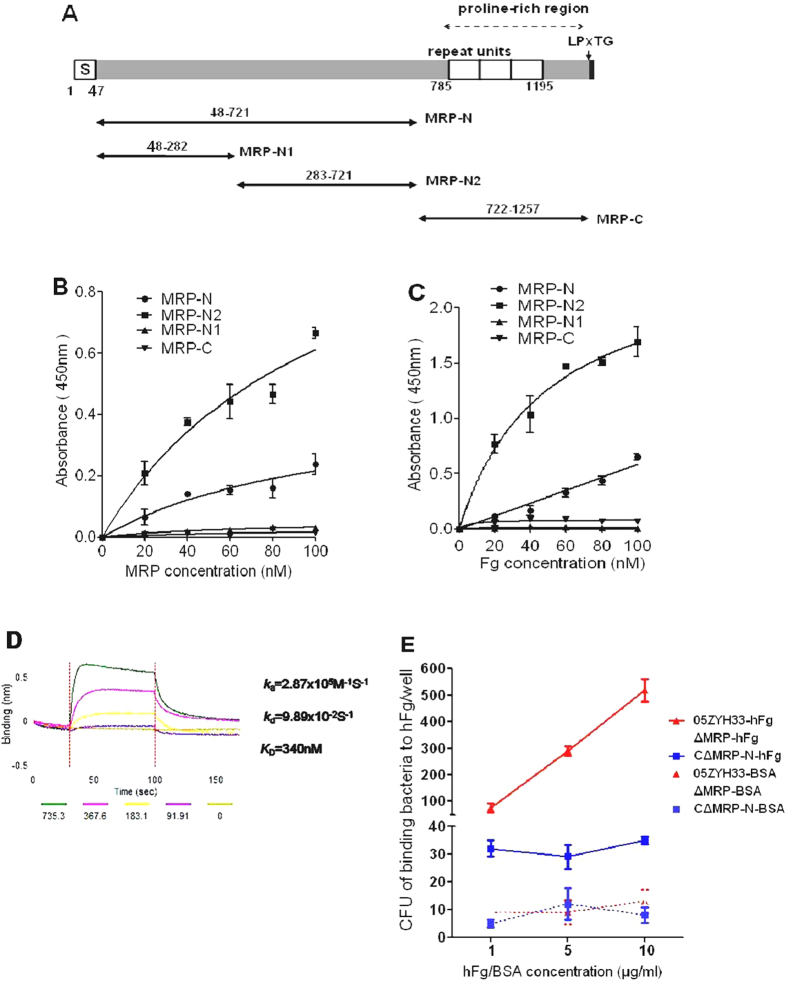
Localization of the hFg-binding region in MRP. (**A**) Schematic of MRP. Arrows indicate the truncated forms of MRP that were tested. *S: signal sequence; LPxTG: cell wall anchoring motif. (**B**) Immobilized Fg (0.25 μg/well) was incubated with the indicated concentrations of truncated hisMRP proteins (_his_MRP-N_48–721_, _his_MRP-N1_100–282_, _his_MRP-N2_283–721_ and _his_MRP-C_722–1257_). Bound proteins were detected with a monoclonal anti-his antibody (1:5000) and an HRP-conjugated anti-mouse antibody (1:10,000). The background values obtained for the control (incubated with PBST containing 1% BSA, but without truncated MRP) were subtracted. (C) Immobilized _his_MRP-N_48–721_, _his_MRP-N1_100–282_, _his_MRP-N2_283–721_ and _his_MRP-C_722–1257_ (100 nM) were incubated with the indicated concentrations of biotinylated hFg, and bound proteins were detected with HRP-conjugated streptavidin (1:8000). The background values obtained for the control (incubated with PBST containing 1% BSA, but without biotinylated hFg) were subtracted. The assays were performed in triplicate, and the mean ± SD is shown. (D) The binding constants of _his_MRP-N were evaluated by BLI. Label-free binding of hFg to immobilized _his_MRP-N was conducted in the Octet, which uses streptavidin-coated biosensors to detect protein:protein interactions via BLI. Baseline-corrected BLI response curves (shown in black, with the lower curve corresponding to a lower concentration of injected analyte) were globally fitted to the 1:1 binding model. Kinetic parameters obtained from the fitting are shown. (E) Binding of *S. suis* to immobilized hFg. The *S. suis* strains WT, ∆MRP and C∆MRP-N (~1 × 10^4^ CFU per well) were incubated with wells pretreated with hFg or BSA (1, 5, 10 μg/ml per well). The values represent triplicate wells and the mean ± SD of the CFU of binding bacteria per well.

PMNs are the first line of defense against bacterial infections in the human blood. In our recent study, hFg enhanced the survival of *S. suis* 2 in a PMN killing assay. MRP was one of two major SsFBPs among the putative SsFBPs identified in that assay^15^. However, the specific binding mechanism remained unclear. The putative functional domain of MRP was not predicted in an analysis of the protein sequence with Interproscan (http://www.ebi.ac.uk/Tools), except for a cell wall attachment consensus motif, LPxTG (Supplementary Fig. 1A). Like most surface proteins of Gram-positive bacteria^23^,^24^, MRP contains a proline-rich repeat sequence (Fig. 1A). To determine the Fg-binding region of MRP, four recombinant fragments of MRP (Fig. 1A) were engineered mainly according to its predicted secondary structure (Supplementary Fig. 1B) by Psipred analysis (http://bioinf.cs.ucl.ac.uk/psipred) as follows: MRP-N (a.a. 48-721, the N-terminus of MRP), MRP-N1 (a.a. 48–282, the α-coil-rich region), MRP-N2 (a.a. 283–721, mainly consisting of β-sheets), and MRP-C (722–1257, a proline-rich region mainly formed by β-sheets). An interaction between recombinant MRP and immobilized hFg was identified for _his_MRP-N (a.a. 48–721) and _his_MRP-N2 (a.a. 283–721) (Fig. 1B), which increased as a function of the protein concentration. We detected no Fg-binding activity for _his_MRP-N1 (a.a. 48–282) or the C-terminal _his_MRP-C (a.a. 722–1257) (Fig. 1B). Similarly, when the system was reversed, immobilized _his_MRP-N and _his_MRP-N2, but not _his_MRP-N1 and _his_MRP-C, showed binding to Fg (Fig. 1C), indicating that a.a. 283–721 encompass the Fg-binding domain of MRP. The binding constants of _his_MRP-N were further evaluated using bio-layer interferometry (BLI). The *K*_*D*_ value for the binding of _his_MRP-N to hFg was 340 nM (Fig. 1D), which was close to the value for the M1 protein in GAS^25^. Additionally, wild-type *S. suis* 05ZYH33 adhered to immobilized hFg were significantly higher than those observed with ∆MRP bacteria or C∆MRP-N (which express only the soluble binding region, MRP-N) (Fig. 1E), indicating that MRP is an important FBP of *S. suis*. These results provide important clues about the function of MRP in *S. suis* 2-induced pathogenesis.

### Identification of the hFg-binding site of MRP

**Figure 2:**
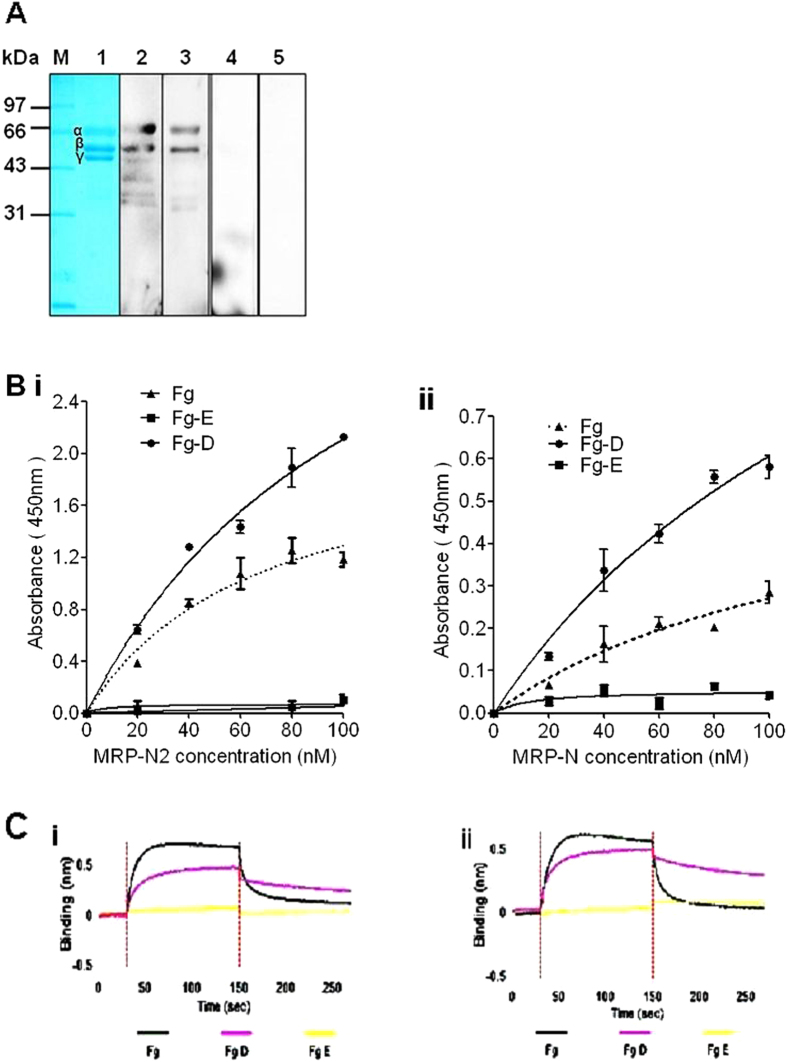
MRP binding to the D fragment of the Fg Aα and Bβ chains. (**A**) Binding of _his_MRP to the hFg Aα and Bβ chains. Purified hFg was separated by SDS-PAGE and stained with Coomassie blue (lane 1) or transferred to PVDF and probed with biotinylated _his_MRP-N (lane 2), _his_MRP-N2 (lane 3), _his_MRP-N1 (lane 4) (20 μg/ml) or PBST (lane 5). The bound proteins were detected with HRP-conjugated streptavidin (1:8000). (**B**) MRP binds to Fg-D, but not Fg-E, as determined by an ELISA-type binding assay. Immobilized Fg/Fg-D /Fg-E (0.25 μg/well) was incubated with the indicated concentrations of _his_MRP-N2 and _his_MRP-N, and bound proteins were detected with anti-his antibody (1:5000) and HRP-conjugated anti-mouse antibody (1:10,000). The background values obtained for the control (incubated with PBST containing 1% BSA, but without truncated MRP) were subtracted. The data show that MRP-N2 (i) and MRP-N (ii) bound to the immobilized Fg (triangles) and Fg-D (circles), but not Fg-E (squares). The assays were performed in triplicate, and the mean ± SD is shown. (**C**) BLI analysis of the binding response curves, which were generated by incubating Fg/Fg-D/Fg-E with immobilized _his_MRP-N2 or _his_MRP-N. Fg-D, but not Fg-E, binds to both _his_MRP-N2 (i) and _his_MRP-N (ii).

Most FBPs of pathogens have specific Fg-binding mechanisms^7^; thus, the region within fibrinogen that is responsible for MRP binding was investigated. Fg consists of subunits D and E, each containing three polypeptide chains (Aα, Bβ and γ). When separated by SDS-PAGE under reducing conditions, fibrinogen appeared as three bands corresponding to the Aα (63.5 kDa), Bβ (56 kDa) and γ (47 kDa) chains (Fig. 2A). Both the Aα and Bβ chains were readily detected when transferred to a polyvinylidene difluoride (PVDF) membrane and were probed with purified biotinylated _his_MRP-N and _his_MRP-N2, but not _his_MRP-N1 (Fig. 2A). To identify the MRP-binding site(s) in Fg, isolated D and E fragments of Fg (Fg-D and Fg-E, respectively) were tested in an ELISA-type binding assay. The data indicate that _his_MRP-N2 (Fig. 2Bi) and _his_MRP-N (Fig. 2Bii) bound well to Fg-D and intact Fg, but did not bind to Fg-E. An analysis of the MRP–Fg-D interaction by BLI further confirmed that Fg-D, but not Fg-E, bound to _his_MRP-N2 (Fig. 2Ci) and _his_MRP-N (Fig. 2Cii), providing insight into a possible mechanism by which MRP alters Fg to allow *S. suis* 2 to evade innate immunity.

### The presence of MRP in culture supernatants and the bacterial cell surface

**Figure 3:**
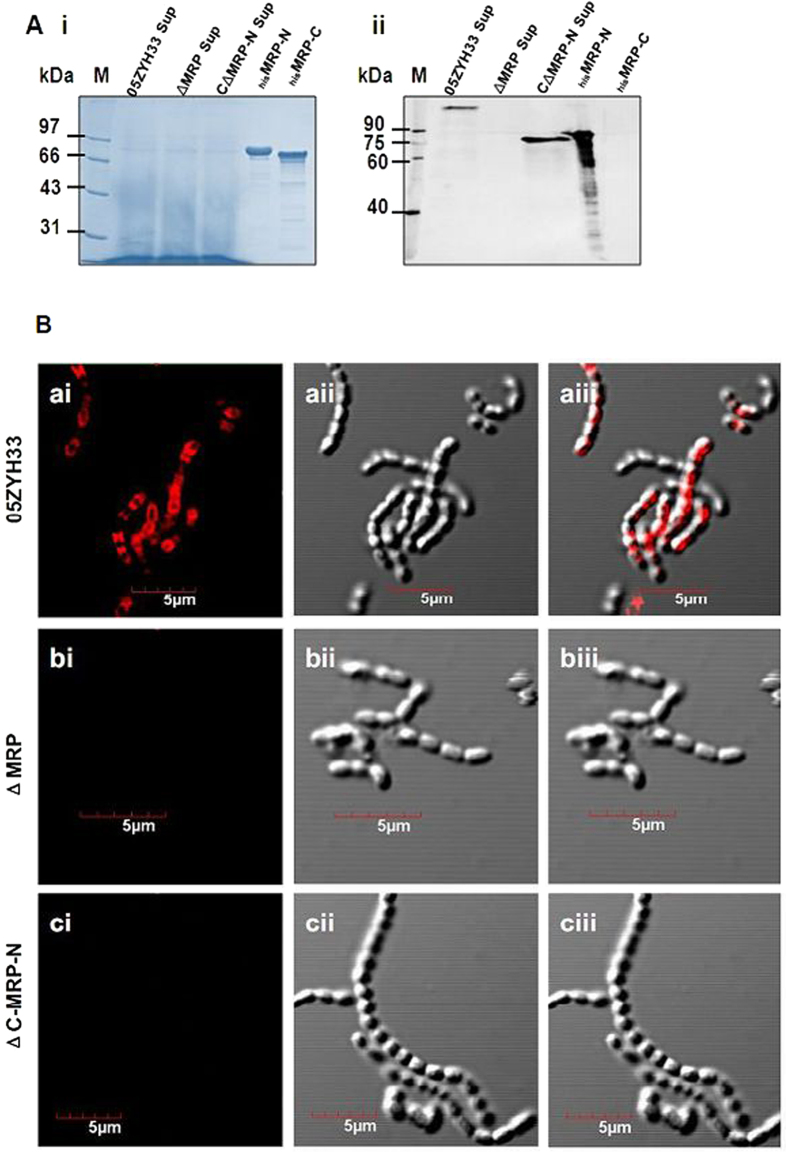
The presence of MRP in the culture supernatant and on the bacterial cell surface. (**A**) The released MRP in the supernatant was detected by western blotting. Samples of the concentrated culture supernatant (25:1, OD_600_ = 0.4) and truncated _his_MRP proteins were separated by SDS-PAGE (Ai) and transferred to a PVDF membrane that was probed with rabbit anti-MRP-N serum (1:400) followed by HRP-conjugated goat anti-rabbit IgG (1:8000). MRP and MRP-N could be specifically detected in the supernatants from WT and C∆MRP-N bacteria, respectively (Aii). The specificity of the anti-MRP-N antibody was evaluated using _his_MRP-N and _his_MRP-C (Aii). (**B**) Visualizing MRP protein on the bacterial surface. The samples of the *S. suis* WT strain 05ZYH33 (ai)-(aiii), the mutant ∆MRP strain (bi)-(biii) and the complementary strain C∆MRP-N that expresses only soluble MRP-N (ci)-(ciii) were washed and stained for MRP-N using rabbit anti-MRP-N IgG and rhodamine (TRITC)-conjugated goat anti-rabbit IgG. For each sample, the merges of the two images are shown in panels (aiii), (biii) and (ciii), respectively.

MRP is a substrate of sortase A in a National Collection of Type Cultures (NCTC) European strain^26^. However, the protein is also detectable in the culture supernatants from serotype 2 strains isolated from European diseased pigs^16^. To further explore the function of MRP in 05ZYH33, the location of MRP was detected in 05ZYH33 cells. Western blotting indicated that MRP was detected in the culture supernatant in the 05ZYH33 strain (Fig. 3A) but was not detected in the mutant strain (Fig. 3A). To elucidate the function of soluble (released) MRP, a complementary strain (C∆MRP-N) was constructed for the soluble expression of MRP-N (which is identified as the hFg-binding region in Fig. 1). Western blotting analysis indicated that MRP-N was present in the supernatant from the C∆MRP-N strain (Fig. 3A). Additionally, the surface location of MRP was visualized using confocal laser scanning microscopy in the WT strain 05ZYH33 (Fig. 3Baiii), but not in the mutant (Fig. 3Bbiii) and complementary strains (Fig. 3Bciii). These data indicated that MRP was released into culture supernatant and that the complementary strain (C∆MRP-N) expressing the secreted MRP-N was successfully constructed.

### MRP facilitates the survival of S. *suis* 2 in human blood in an α_X_β_2_ integrin-dependent manner

**Figure 4:**
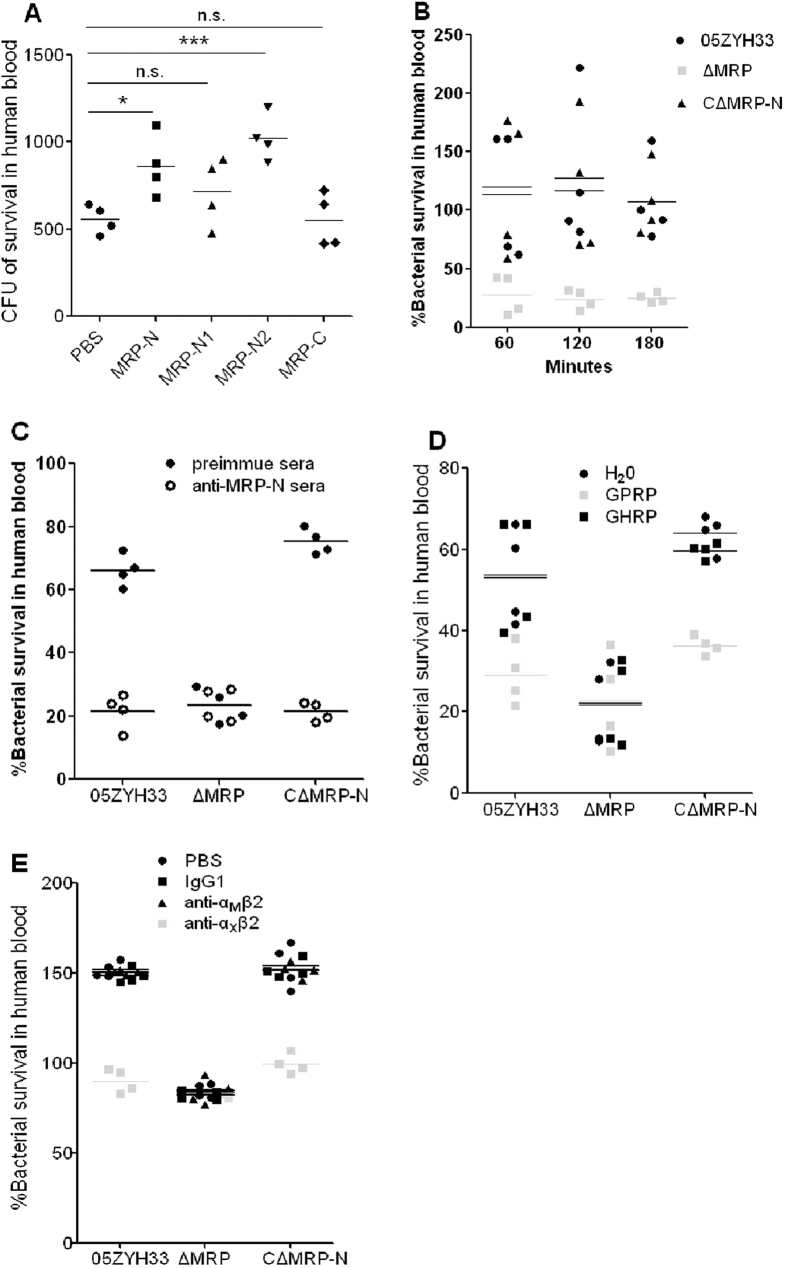
MRP facilitates *S. suis* 2 survival in human blood in an α_X_β_2_ integrin-dependent manner. (**A**) Incubation of ∆MRP bacteria with the MRP-binding domain (1.0 mM) increased bacterial survival in human blood after 60 min. *P < 0.05, ***P < 0.001; n.s., no significance. (**B**) MRP contributes to the survival of *S. suis* 2 in human blood at the measured time points (60, 120 and 180 min). Significant differences were detected between the WT and ∆MRP groups (*P* < 0.001 at 60 min; *P* < 0.05 at 120 min and 180 min) and between the C∆MRP-N and ∆MRP groups (*P* < 0.001 at 60 min; *P* < 0.05 at 120 min and 180 min). (**C**) Antibodies against the MRP-N-binding region decrease *S. suis* viability in human blood. Bacteria were pretreated with preimmune (control) or anti-MRP-N serum from rabbits immunized with MRP-N (1:5 dilution). Significant differences were found between preimmune and anti-MRP-N sera in the WT (*P* < 0.001) and C∆MRP-N (*P* < 0.001) groups, but not in the ∆MRP group (*P* > 0.05). (**D**) Viability of *S. suis* 2 in human blood in the presence or absence of GPRP (50 μg/ml) and GHRP (50 μg/ml). Significant differences were found between GPRP treatment and other treatments in the WT (*P* < 0.05) or C∆MRP-N (*P* < 0.001) groups but not in the ∆MRP group (*P* > 0.05). (**E**) Viability of *S. suis* 2 in human blood as determined by a bactericidal assay after treatment with the specific anti-α_M_β_2_ antibody (10 μg/ml), the specific anti-α_x_β_2_ antibody (10 μg/ml) or their isotype control mouse IgG1 (10 μg/ml). Significant differences were found between anti-α_x_β_2_ treatment and other treatments in the WT (*P* < 0.001) and C∆MRP-N (*P* < 0.001) groups but not in the ∆MRP group (*P* > 0.05). In Figs. A–E, each symbol represents an individual human blood sample. In Figs. A, C, D and E, the horizontal lines indicate the mean for each group. In Figs. A, D and E, post hoc tests were used for statistical analysis. In Fig. C, an unpaired Student’s *t* test was used. In Fig. B, the horizontal lines indicate the median for each group, and a Mann-Whitney test was used.

To investigate the role of MRP in immune evasion in human blood, the survival of *mrp*-deficient mutants (∆MRP) was evaluated after the addition of _his_MRP truncations. Interestingly, the viability of strain ∆MRP in human blood was increased by the exogenous addition of _his_MRP-N or _his_MRP-N2, but not _his_MRP-N1 or _his_MRP-C (Fig. 4A). Therefore, it is possible that the biological effects of MRP are exerted upon its release into the extracellular milieu. As expected, C∆MRP-N rescued the survival defect of the mutant ∆MRP bacteria in the blood, bringing the survival to levels similar to WT (Fig. 4B). Additionally, in a specific antibody-blocking assay, antiserum against MRP-N decreased the WT and C∆MRP-N bacterial survival, but not to ∆MRP in human blood (Fig. 4C), suggesting that MRP could be a promising candidate for antibody-based therapies for *S. suis* induced-sepsis. These results indicate that soluble (released) MRP is responsible for immune evasion by *S. suis* in human blood.

The mechanistic basis of MRP in immune evasion by *S. suis* 2 was further investigated in a bactericidal assay using a peptide derived from the Aα chain of Fg (Gly-Pro-Arg-Pro, GPRP), which acts as a β_2_ integrin antagonist and has been shown to impair the M1-Fg complex-induced aggregation of PMNs^27^. A second peptide, Gly-His-Arg-Pro (GHRP), which does not have this effect^27^, was also tested as a control. GPRP decreased the growth of WT and C∆MRP-N bacteria in human blood (Fig. 4D), while no effects were observed for the ∆MRP bacteria. The control peptide GHRP or H_2_O had no effect on the survival of WT, ∆MRP and C∆MRP-N in human blood, suggesting that the β_2_ integrins on PMNs were involved in this process. α_X_β_2_ and α_M_β_2_ are general integrins expressed by phagocytes; neutrophils interact with hFg through the β_2_ family of integrins, with the N-terminal portion of the Aα chain of Fg binding to α_X_β_2_^28^ while the C-terminal portion of the γ chain binds to α_M_β_2_ integrins^29^. To determine which type of β_2_ integrin mediates the formation of MRP-hFg complexes for immune evasion by *S. suis* 2, monoclonal antibodies against the α_M_ (clone CBRM1/5) and α_x_ (clone BU15) integrin subunits and an isotype control mouse IgG1 were used in blocking assays. Like GPRP, the antibody against the α_x_ integrin subunit decreased the survival of WT and C∆MRP-N, but not ∆MRP bacteria in human blood, while there was no interference detected using the antibody against α_M_ or the control IgG1 (Fig. 4E).

These results suggest that the N-terminus of MRP (MRP-N) might interact with hFg and exert some effects on PMNs via α_X_β_2_ integrin crosslinking to improve the survival of *S. suis* 2 in human blood.

### The soluble (released) MRP was involved in the immune evasion of *S. suis* 2 by interacting with hFg

**Figure 5:**
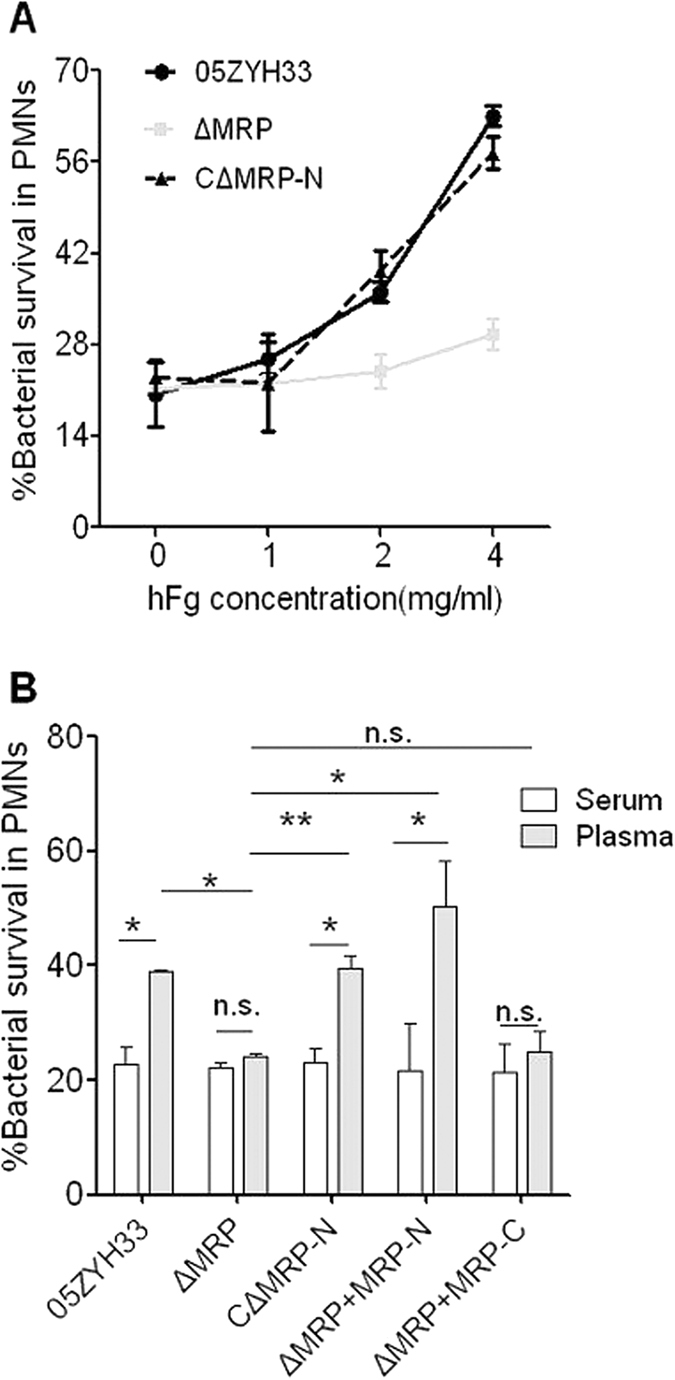
The MRP–hFg interaction was involved in the immune evasion of *S. suis* 2. (**A**) The MRP-hFg interaction enhanced the survival of *S. suis* 2 in serum supplemented with hFg in PMN killing assays. The WT, C∆MRP-N and ∆MRP strains were co-incubated with PMNs at an MOI of 1:15 (PMN:bacterium) in 50% serum without or with hFg (1–4 mg/ml) for 60 min. The mutant ∆MRP bacteria had reduced viability compared to the WT (*P* < 0.01 at 2 mg; *P* < 0.001 at 4 mg) and C∆MRP-N strains (*P* < 0.01 at 2 mg; *P* < 0.001 at 4 mg) in serum containing hFg at physiological concentrations (2–4 mg/ml), but not in serum alone (*P* > 0.05) or in 1 mg/ml hFg (*P* > 0.05). (**B**) The MRP-hFg interaction increased the survival of *S. suis* 2 in plasma in PMN killing assays. The WT, C∆MRP-N, and ∆MRP strains and the ∆MRP strain with added truncated MRP were incubated with PMNs at an MOI of 1:15 (PMN:bacterium) in 50% serum or plasma isolated from the same healthy donors for 60 min. In Figs. A and B, the percentage of surviving *S. suis* 2 indicated was calculated as follows: (CFU_PMN+_/CFU_PMN−_) × 100%. The data are expressed as the means ± SD of three independent experiments, and unpaired Student’s *t* tests were used for statistical analysis. *P < 0.05; **P < 0.01; n.s. (no significance).

PMN-mediated innate immunity is the first line of defense against bacterial pathogens in human blood. To verify the hypothesis that MRP improves the immune evasion of *S. suis* 2 by interacting with hFg, a simplified model of whole blood, a PMN killing assay, was performed in the presence of fresh serum, fresh plasma or fresh serum supplemented with hFg (the serum and plasma were isolated from the same donor). Interestingly, as in the whole blood, the mutant ∆MRP bacteria had reduced viability compared to the WT bacteria in serum containing hFg at physiological concentrations (2–4 mg/ml) (Fig. 5A), and C∆MRP-N rescued the viability of ∆MRP to the level of WT in serum with hFg, but not in serum alone (Fig. 5A). Similarly, ∆MRP bacteria had reduced viability compared to WT and C∆MRP-N bacteria in plasma (Fig. 5B), but not in serum alone; additionally, supplementation of ∆MRP bacteria with MRP-N, but not MRP-C, rescued their viability in plasma but had no effect in serum alone (Fig. 5B). These results indicate that the N-terminus of MRP (MRP-N) is responsible for the immune evasion of *S. suis* by interacting with hFg.

### The MRP-hFg interaction results in the integrin-triggered aggregation of PMNs and PMN exhaustion

**Figure 6:**
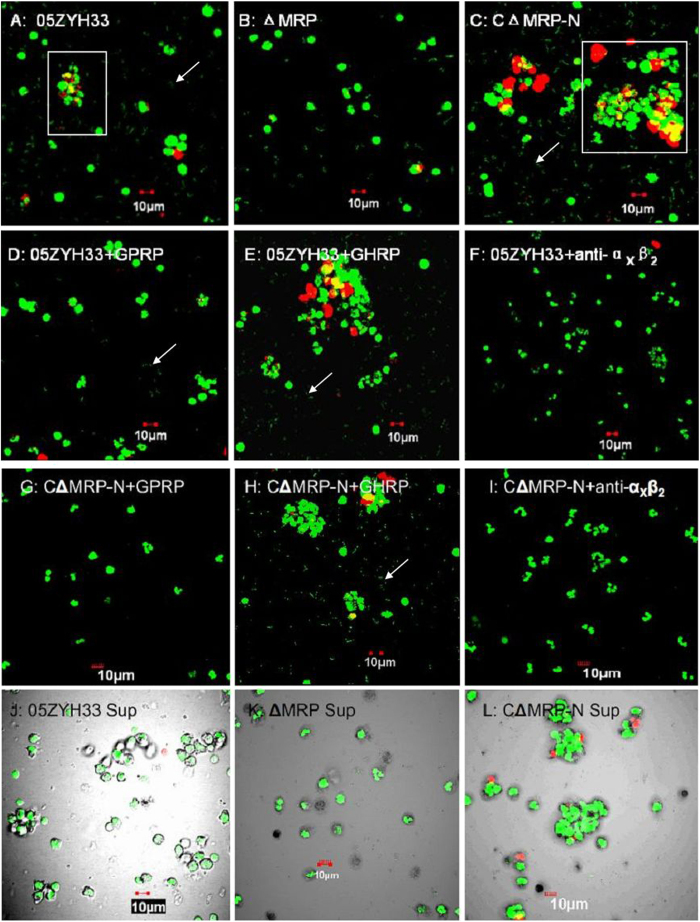
PMN aggregation via an MRP-hFg interaction through α_x_β_2_ integrin crosslinking. PMNs incubated with *S. suis* at an MOI (PMN:bacterium) of 1:15 in 50% serum supplemented with hFg or serum alone for 60 min were analyzed by confocal laser scanning microscopy. Dead and viable PMNs/bacteria were stained with PI (red staining) and SYTO 9 (green staining), respectively. The extracellular viable bacteria are indicated by a white arrow. The regions enclosed by white boxes are shown enlarged in Fig. S1. PMNs were incubated with WT (**A**), ∆MRP (**B**) or C∆MRP-N (**C**) bacteria; PMNs pretreated with GPRP (**D)**, GHRP (**E**) or anti-α_x_β_2_ antibody **(F**) were incubated with WT bacteria; PMNs pretreated with GPRP (**G**), GHRP (**H**) or anti-α_x_β_2_ antibody (**I**) were incubated with C∆MRP-N bacteria; PMNs were incubated with the supernatants from WT (**J**), ∆MRP (**K**) and C∆MRP-N (**L**) bacteria. The bars are as indicated in Fig. 6. GPRP, Gly-Pro-Arg-Pro; GHRP, Gly-His-Arg-Pro; anti-α_x_β_2_, monoclonal antibody against α_x_ integrin subunits; Sup, supernatant.

The effects of the MRP–hFg interaction on PMNs in the PMN killing assay were further investigated by confocal laser scanning microscopy. The WT (Fig. 6A) and C∆MRP-N (Fig. 6C) strains induced PMN aggregation in serum supplemented with hFg; however, infection by either the ∆MRP strain (Fig. 6B) or the WT strain in serum alone had no effect (data not shown), suggesting that the aggregation was caused by the MRP–hFg interaction. Moreover, dead bacteria and PMNs (red staining by propidium iodide [PI]) were the principal constituents of these aggregates induced by the WT or C∆MRP-N strains (Fig. 6A,C and their magnified images in Supplementary Fig. 2), and greater numbers of green extracellular viable bacteria (green staining by SYTO9) were observed in these groups (white arrows in Fig. 6A,C and in Supplementary Fig. 2) than with the ∆MRP strain (Fig. 6B). Because soluble MRP enhanced the survival of *S. suis* 2 in PMN killing assays by interacting with hFg, the supernatants were assessed to determine whether they could induce PMN aggregation. As expected, the WT and C∆MRP-N-supernatants had a similar effect on PMNs in the presence of serum supplemented with hFg (Fig. 6J,L), but the ∆MRP supernatant did not (Fig. 6K). These results suggest that soluble (released) MRP induces PMNs aggregation and death by interacting with hFg.

Furthermore, GPRP, but not GHRP, inhibited the aggregation induced by the WT (Fig. 6D,E) and C∆MRP-N (Fig. 6G,H) strains, as did a monoclonal antibody against α_X_ integrin subunits (Fig. 6F,I). These results were consistent with the bactericidal assay in Fig. 5A,B, suggesting that the MRP-hFg interaction results in the α_X_β_2_ integrin-triggered aggregate formation of PMNs and reduces the capacity of PMNs to clear *S. suis* in the blood by phagocytosis.

**Figure 7:**
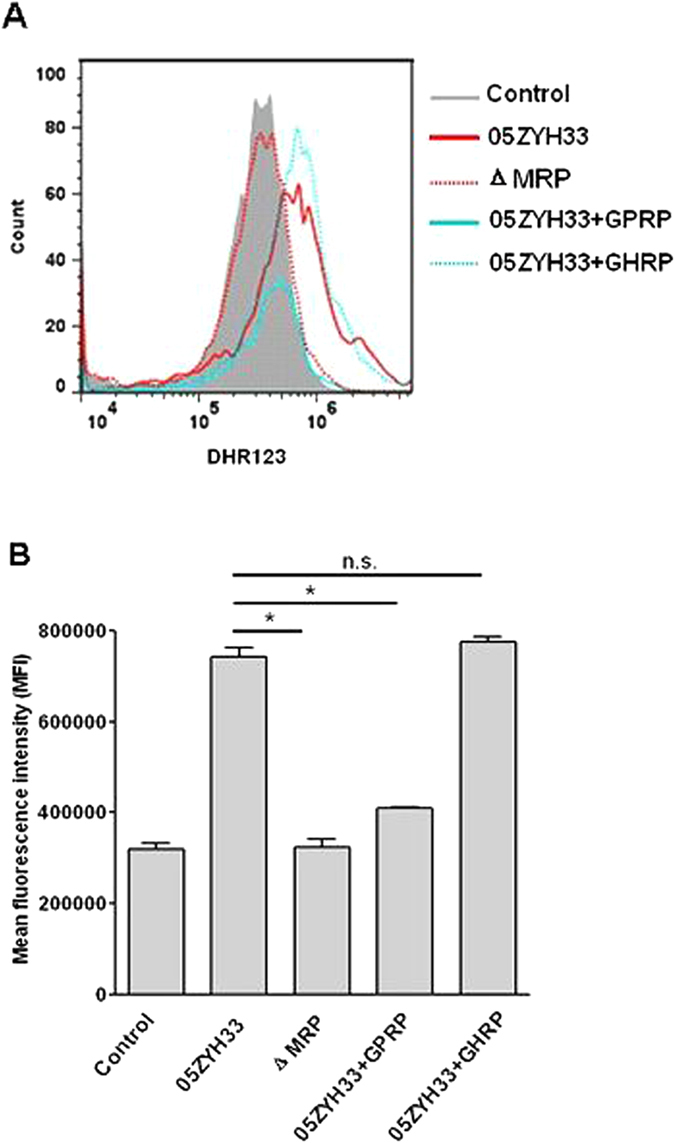
Flow cytometric analysis of oxidative activity in the PMN killing assay. (**A**) Representative histograms for fluorescence-activated cell sorting (FACS) analysis. (**B**) The MFI of DHR123 was measured. In the PMN killing assay, the PMNs were stimulated with the WT or ΔMRP strains in the absence or presence of GPRP or GHRP. PMNs without *S. suis* stimulation served as the control. DHR123 (1 μM) was used to detect oxidative activity. The data are expressed as the means ± SD of three replicates. *P < 0.05; n.s., no significance.

To further analyze PMN activation by *S. suis*, dihydrorhodamine (DHR)123, a fluorogenic substrate of active oxygen, was used to detect oxidative activity. The mean fluorescence intensity (MFI) as determined by flow cytometry in PMNs treated with the WT strain was twice that of PMNs treated with ∆MRP. The MFI of PMNs was 2-fold lower in the presence of GPRP relative to GHRP upon treatment with the WT strain opsonized in hFg-supplemented serum (Fig. 7), but not in serum alone (data not shown). These results suggest that the MRP–hFg interaction results in the excessive activation and exhaustion of PMNs via a β_2_ integrin-dependent mechanism.

## Discussion

*S. suis* is an emerging pathogen and a leading cause of skin wounds and bloodstream, brain, and soft tissue infections^30^,^31^. The most common clinical manifestations of infection are sepsis and meningitis^30^, which are often accompanied by bacteremia, as is the case for *Streptococcus pneumoniae*- and *Neisseria meningitidis*-associated meningitis^32^. Outbreaks of *S. suis* 2 infection in China have highlighted the importance and relatively high frequency of a severe sepsis syndrome that shares features with toxic shock syndrome and is associated with high mortality^1^,^3^. However, little is known of the mechanism of *S. suis* survival in human blood, and only a few virulence factors, such as Fhb^24^ and SLY^33^, are known to enable pathogens to evade the host immune response by inhibiting complement activation.

MRP is often reported as an epidemic infection marker in Europe and Asia^19^,^20^,^21^. Although this protein was reported not to be a critical virulence factor in an intranasal infection model in Anthony piglets^34^, the real contribution of MRP to bacterial virulence needs to be identified in a rational infection model because different infection models might lead to different conclusions^35^. For example, the Canadian virulent strain 89-1591 (MRP^-^) was reported as avirulent by Rasmussen *et al.*^36^ but was shown to be virulent in experimental models applied by other researchers^37^. Several North American virulent strains were reported to be MRP^−^ genotype strains^38^. However, most North America virulent strains (MRP^-^) belong to ST25 strains, which were different from ST7 strains based on multilocus sequence typing^39^. The highly virulent Chinese strain 05ZYH33 used in this study belongs to ST7 strains which exhibit strong invasiveness and high pathogenicity, has resulted in the description of a new disease form of streptococcal toxic shock syndrome (STSS) and a putative pathogenicity island (termed 89 K)^31^. We believe that the real contribution of MRP to different strains need to consider the background of strains.

PMNs are the first line of defense against bacterial infections in human blood; in our recent study, MRP enhanced the survival of *S. suis* 2 in PMN killing assays by interacting with hFg^15^. However, the specific mechanism of this enhancement needed to be clarified. In this study, MRP was identified as a novel FBP of *S. suis* 2 that showed high binding affinity to hFg, suggesting that the MRP–hFg interaction may be critical for MRP function. The present study revealed that binding occurs via the interaction of the N-terminus of MRP (a.a. 283–721) and the D fragment of Fg, similar to that observed in the adhesins of other gram-positive bacteria^7^,^11^. Nonetheless, there are some notable differences from other FBPs: first, the binding region of MRP has no sequence homology to other known FBPs, which share a common domain architecture that enables fibrinogen binding through a “dock, lock, and latch” (DLL) mechanism^40^,^41^,^42^,^43^. Second, in contrast to other FBPs that bind the Aα, Bβ or γ chain singly, MRP displayed specific binding to both the Aα and Bβ chains in a manner similar to Lysin, an FBP of *Streptococcus mitis*^44^, but different from that of the M1 protein, which binds to coiled coils in the β and γ chains of hFg^45^. Studies have been initiated to determine the crystal structure of the MRP–hFg complex, which can reveal the details of this interaction.

An intriguing observation was that the soluble MRP-hFg interaction enhanced the survival of *S. suis* 2 in human blood and induced the aggregation and hyperactivation of PMNs via the α_X_β_2_ integrin receptor, which was independent of complement inhibition (data not shown). However, M proteins are important virulence factors for GAS that inhibit complement deposition on the bacterial surface via the binding of hFg to the surface M proteins^8^,^46^. Although the soluble (released) M1 induced PMN aggregation and HBP release and was blocked by the β_2_ integrin antagonist GPRP^25^, it was not determined which member of the β_2_ integrin subfamily was involved in the crosslinking of the M1–hFg complex and the immune evasion of GAS. Despite hFg being a physiologically relevant ligand for α_M_β_2_, the integrin engagement of hFg is critical for leukocyte function and innate immunity *in vivo*^47^. α_X_β_2_, but not α_M_β_2_, was involved in the excessive activation of PMNs induced by the MRP–hFg complex in the present study. Although other FBPs, including M1^25^ and FOG^10^, induce PMN aggregation, the specific receptor on PMNs that mediates crosslinking to the FBP/hFg complexes and the relationship of this process to immune evasion by pathogens remained unclear.

In summary, MRP is a known infection marker of *S. suis* 2 in most Chinese and European clinical isolates, but the role of MRP in the evasion of PMN-mediated innate immunity by *S. suis* remained unclear and controversial. Here, we showed that the N-terminus of MRP (a.a. 283–721) binds to both the Aα and Bβ chains of the D fragment in hFg. Moreover, the MRP-hFg interaction aggregates PMNs via the α_X_β_2_ integrin receptor and promotes pathogen survival in host blood. These data shed new light on the function of MRP in the *S. suis*-host interaction. More objective consideration of strains and infection models is needed to reevaluate the contribution of MRP to the virulence of ST-7 strains in China and ST-1 strains in Europe.

## Methods

### Human blood, serum and plasma

The healthy donors who provided the serum and plasma in this study provided written informed consent in accordance with the Declaration of Helsinki. Approval was obtained from the Institutional Medical Ethics Committee of the Academy of Military Medical Sciences. The human blood, serum and plasma used in this study were obtained in accordance with the approved guidelines.

### Bacterial strains and growth conditions

The highly virulent *S. suis* 2 strain 05ZYH33, originally isolated from a deceased STSS patient following an outbreak in Sichuan, China in 2005, was used in this study. *S. suis* 2 strains were cultured as previously described^24^. The bacterial strains and plasmids used in this study are listed in Supplementary Table 1.

### Recombinant expression and purification of MRP

To determine the binding region of MRP, four recombinant fragments of MRP (Fig. 1A) were engineered mainly according to its predicted secondary structure (Supplementary Fig. 1B) as follows: MRP-N (a.a. 48–721, the N-terminus of MRP), including MRP-N1 (a.a. 48–282, the α-coil-rich region), MRP-N2 (a.a. 283–721, mainly consisting of β-sheets) and MRP-C (722–1257, a proline-rich region mainly consisting of β-sheets). The oligonucleotide primers and expression vector used for MRP-N, MRP-N1, MRP-N2 and MRP-C in this study are listed in Supplementary Table 1. All of the above ORFs were cloned into the pET-28a vector, and the proteins were expressed and purified by Ni-chelating chromatography (GE Healthcare) according to the manufacturer’s recommendations.

### Preparation of anti-MRP-N sera and IgG

To generate polyclonal anti-MRP-N antibodies, the recombinant MRP-N proteins were injected subcutaneously into two female rabbits as previously described^20^. All experiments on live vertebrates in this study were approved by the Institutional Medical Ethics Committee of the Academy of Military Medical Sciences; the permit number of all animal work was SCXK-(JUN) 2013–018. All experiments on live vertebrates were performed in accordance with the relevant guidelines and regulations^20^. The anti-MRP-N IgG in the antiserum was purified using a HiTrap Protein G HP column (GE Healthcare).

### ELISA-type binding assay

To detect the binding of MRP to immobilized hFg, 96-well plates were coated overnight at 4 °C with 100 μl of 2.5 μg/ml Fg/Fg-D/Fg-E (Merck). After blocking with 3% BSA (bovine serum albumin) in PBST (phosphate-buffered saline with 0.05% Tween-20), recombinant MRP proteins were added and incubated for 1 h at room temperature. Bound MRP was detected by incubation with an anti-his monoclonal antibody (1:5000, Sigma) and HRP (horseradish peroxidase)-conjugated anti-mouse antibodies (1:10,000; Santa Cruz). The binding was quantified by measuring the absorbance at 450 nm. Background values obtained for the control (incubated with PBST containing 1% BSA, without truncated MRP) were subtracted. To detect the binding of hFg to immobilized MRP, 96-well plates were coated overnight at 4 °C with 100 μl of 100 nM truncated MRP. After blocking with 3% BSA in PBST, biotinylated hFg was added, and the plates were incubated for 1 h at room temperature. Bound hFg was detected by incubation with HRP-conjugated streptavidin (1:8000; GE Health). Background values obtained for the control (incubated with PBST containing 1% BSA, without biotinylated hFg) were subtracted. The assays were performed in triplicate, and the mean ± SD is shown.

### BLI analysis of the MRP-hFg interactions

All BLI experiments were performed in PBST on a BLItz system (ForteBio, Inc.) as previously described^48^. Streptavidin-coated biosensors with immobilized biotinylated MRP-N proteins were exposed to different concentrations of hFg, Fg-D, or Fg-E. The association and dissociation rate constants (*k*_*a*_ and *k*_*d*_, respectively) for the interactions were derived from curve fitting and were used to calculate the dissociation constant, *K*_*D*_ (*K*_*D*_ = *k*_*d*_/*k*_*a*_).

### Binding of S. suis to immobilized fibrinogen

The binding of *S. suis* to immobilized fibrinogen was detected as previously described^43^. The 96-well plates were coated overnight at 4 °C with 100 μl of purified fibrinogen (1, 5, 10 μg/ml) or BSA (1, 5, 10 μg/ml) and were then washed four times with PBS. Overnight cultures of the WT, CΔMRP-N and ΔMRP strains were harvested by centrifugation and then adjusted to almost equal the original inocula (~10^5^ CFU [colony-forming units]/ml) in PBS. The wells, which were pretreated with hFg/BSA, were then incubated with 100 μl of *S. suis* suspension for 30 min at 37 °C. The wells were washed four times with PBS to remove unbound bacteria and were then treated with 100 μl of trypsin (2.5 mg/ml) for 10 min at 37 °C to release the attached bacteria. The number of bound bacteria was determined by plating serial dilutions of the recovered bacteria onto THB agar plates.

### Generation of the ∆MRP mutant and the complementary CΔMRP-N strains

The *mrp* mutant was obtained from the 05ZYH33 WT strain (accession number YP_001198119) by in-frame deletion of the *SSU05_0753* gene, as previously described^15^,^49^. In the present study, for complementation assays, we selected MRP-N, but not the entire MRP sequence for cloning because MRP exists as a soluble protein in the supernatant, and MRP-N (a.a. 48–721) was the hFg-binding region. Furthermore, it was difficult to construct a complementary strain expressing the entire MRP protein. The upstream promoter and a DNA fragment encoding MRP-N without the LPxTG motif were amplified using the primers CΔMRP-N-F/CΔMRP-N-R (Supplementary Table 1). The amplicon was subsequently cloned into the *E. coli*-*S. suis* shuttle vector pAT18, the recombinant plasmid pAT18::*mrp*-N was transformed into the ΔMRP strain, and the CΔMRP-N strain was screened on THB agar with selective pressure from Em. Western blotting and confocal laser scanning microscopy analysis were used to confirm the soluble expression of MRP-N in CΔMRP-N bacteria.

### Bactericidal and PMN killing assays

The bactericidal assay was used to compare the growth of the WT, CΔMRP-N and ΔMRP strains in human blood as described previously^24^. Briefly, diluted strain cultures (50 μl at 2 × 10^4^ CFU/ml) were combined with fresh human blood (450 μl), and the mixtures were rotated at 37 °C; the samples were treated with 1% saponin, diluted, and plated onto blood agar. The percentage of live bacteria was subsequently calculated as (CFU on plate/CFU in original inoculum) × 100%. In the integrin-blocking assay, GPRP (50 μg/ml), GHRP (50 μg/ml), monoclonal antibodies against α_M_ (clone CBRM1/5, eBioscience, 10 μg/ml), α_x_ integrin subunits (clone BU15, Acris Antibodies, 10 μg/ml) and the isotype control mouse IgG1 antibody (eBioscience, 10 μg/ml) were preincubated with the blood for 15 min at room temperature. In the PMN killing assay, PMNs were infected with *S. suis* 2 at a multiplicity of infection (MOI) of 1:15 (PMN:bacterium) in 50% non-immune human serum and were centrifuged at 380 × *g* for 5 min at 4 °C. The plates were then incubated at 37 °C in 5% CO_2_. Samples were taken at 60 min and analyzed immediately as described for the human blood. The colonies were counted, and the percentage of surviving *S. suis* 2 bacteria was calculated as follows: (CFU_PMN+_/CFU_PMN−_) × 100%. GPRP and GHRP were dissolved in H_2_O.

### Confocal laser scanning microscopy analysis

The LIVE/DEAD viability kit (Invitrogen) was used to determine the viability of PMNs and *S. suis*. In the PMN killing assay, a mixture of PMNs and bacteria was stained with PI and SYTO 9 and then spotted onto Petri dishes (World Precision Instruments Inc.) using cover slips placed over the liquid to stabilize the cells. The dead (PI, red staining) and viable (SYTO 9, green staining) PMNs/bacteria were then visualized by XYZ scanning using an FV1000 confocal laser scanning microscope (Olympus).

Visualization of MRP proteins on the bacterial surface was performed as previously described^50^. Briefly, an aliquot of streptococci (6 × 10^6^ CFU/mL) was added to rabbit anti-MRP-N IgG (10 μg/ml), incubated for 45 min on ice and washed. Three hundred microliters of the resuspension was then incubated with rhodamine (TRITC)-goat anti-rabbit IgG (Santa Cruz) (0.3 mg/ml) for 45 min on ice. The bacteria were then washed with PBS and spotted onto CITOGLAS slides (SHITAI). The slides were then analyzed with an OLYMPUS FV1000 confocal scanning microscope.

## Additional Information

**How to cite this article**: Pian, Y. *et al.* Binding of Human Fibrinogen to MRP Enhances *Streptococcus suis* Survival in Host Blood in a α_X_β_2_ Integrin-dependent Manner. *Sci. Rep.* **6**, 26966; doi: 10.1038/srep26966 (2016).

## Supplementary Material

Supplementary Information
