# Supplementary material for: Binding of Human Fibrinogen to MRP Enhances Streptococcus suis Survival in Host Blood in a αXβ2 Integrin-dependent Manner
Source: Sci Rep. 2016 May 27;6:26966. doi: 10.1038/srep26966 (PMC4882601; doi:10.1038/srep26966)
Supplement: Supplementary Information [file srep26966-s1.pdf]

Binding of Human Fibrinogen to MRP Enhances *Streptococcus suis* Survival in Host Blood via a $\alpha_X\beta_2$ integrin-dependent manner

Yaya Pian[1,2], Xueqin Li[1], Yuling Zheng[1], Xiaohong Wu[1], Yuan Yuan[1], Yongqiang Jiang[1]

[1] State Key Laboratory of Pathogen and Biosecurity, Beijing Institute of Microbiology and Epidemiology, Beijing 100071, China;

[2] Institute of Biophysics, Chinese Academy of Sciences, Beijing 100101,China;

Corresponding author: Dr. Yuan Yuan and Pro. Yongqiang Jiang

State Key Laboratory of Pathogen and Biosecurity, Beijing Institute of Microbiology and Epidemiology, Beijing 100071

Tel : (86-10) 66948487. Fax: (86-10) 66948637.

E-mail: miniminiyuan@163.com (Y.Y.) or jiangyq@nic.bmi.ac.cn (Y.Q.J.)

Key Words: *Streptococcus suis*; MRP; human fibrinogen; $\alpha X\beta 2$ integrin; human blood

Running Title: MRP of *Streptococcus suis*

Supplementary Table 1. Bacterial strains and plasmids used in this study.

Strain or plasmid	Description[a] or Sequence	Source, reference, PCR products
Strains	Description	
E. coli DH5α	Host for cloning vector	In this lab
05ZYH33	Virulent Chinese *S. suis* serotype 2 isolate	In this lab
ΔMRP	Gene *mrp* knockout mutant strain; Cm^R	reference[15]
CΔMRP-N	Complemented strain of ΔMRP; Cm^REm^R	This study
Plasmids		
pET-28a	Expression vector, lacZ, Kan^R	TaKaRa
pAT18	The complemented expression vector, Em^R	In this lab
pAT18:: *mrp-N*	pAT18 containing the intact *mrp-N* gene and its upstream promoter; Cm^REm^R	This study
Primers	Sequence[b] (5′–3′)	
MRP-N-F	CGC**GGATCC**GATGAAACTGTTGCTTCATCA	The ORF of MRP-N
MRP-N-R	CCG**CTCGAG**CTATAATTTCTGATCCAAGTCGG	
MRP-N1-F	CCGC**GGATCC**GATGAAACTGTTGCTTCATCA	The ORF of MRP-N1
MRP-N1-R	ACCG**CTCGAG**CTATGTTGACGAATCATTATCC	
MRP-N2-F	CGC**GGATCC**ACTGCTGTTTTAGCAGAGTTG	The ORF of MRP-N2
MRP-N2-R	CCG**CTCGAG**CTATAATTTCTGATCCAAGTCGGT	
MRP-C-F	CGC**GGATCC**GCTTCCATCACTTTTGAAGGC	The ORF of MRP-C
MRP-C-R	CCG**CTCGAG**CTATTGAGCTTTACCTGAAGCGGT	
CΔMRP-N-F	CCG**GAATTC**ATAATGTATTTTTGAGGAATTTTAT	The ORF of MRP-N and its upstream promoter
CΔMRP-N-R	CG**GGATCC**TTATAATTTCTGATCCAAGT	

NOTE.[a] Cm^R, chloramphenicol resistant; Em^R, erythromycin resistant; Kan^R, kanamycin resistant; [b] The underlined sequences are the restriction sites

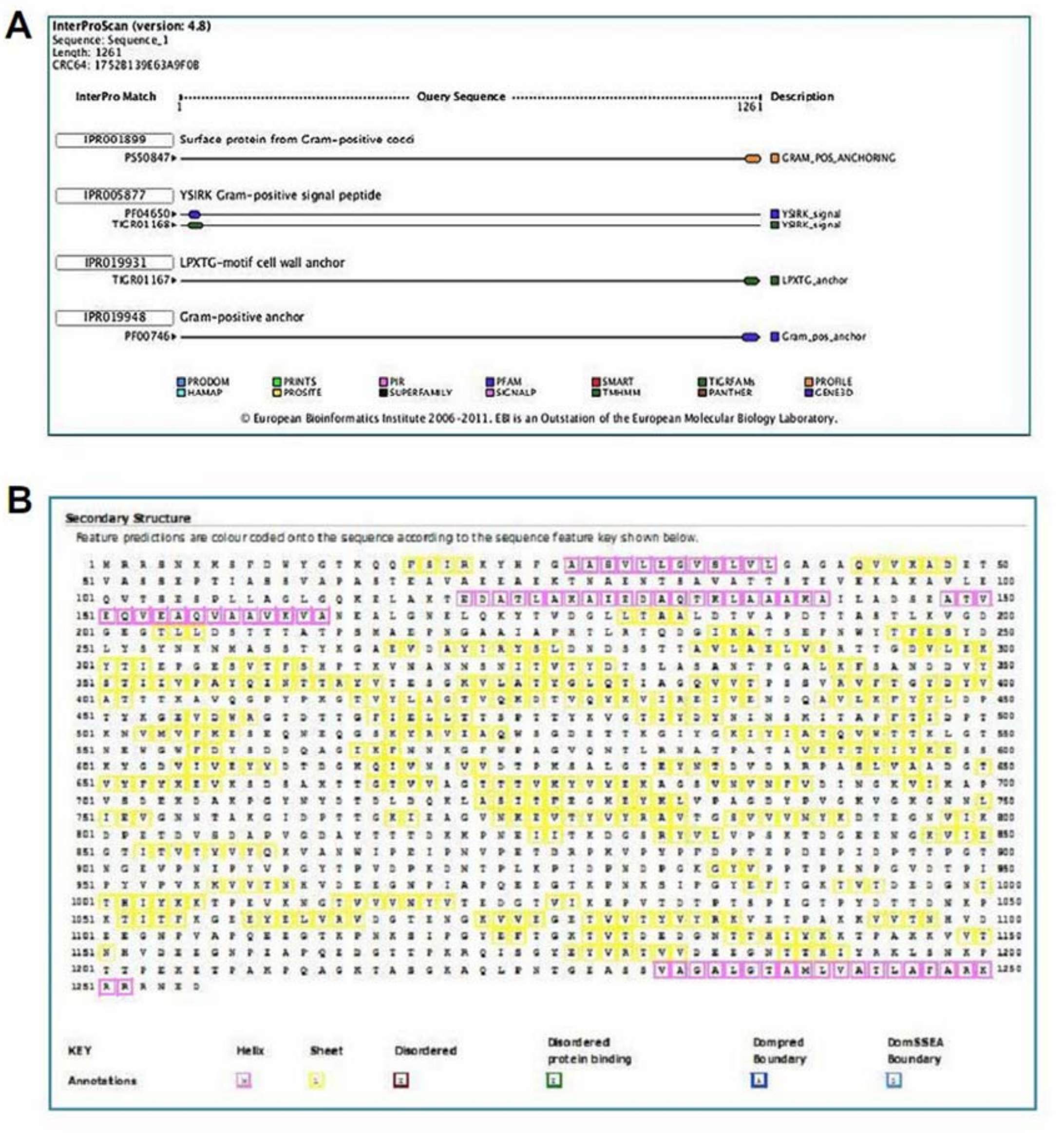

Supplementary Figure 1. Bioinformatics-based analysis of MRP. (A). The MRP putative domain predicted by Interproscan software (http://www.ebi.ac.uk/Tools). (B). The secondary structure of MRP analyzed by Psipred software (http://bioinf.cs.ucl.ac.uk/psipred).

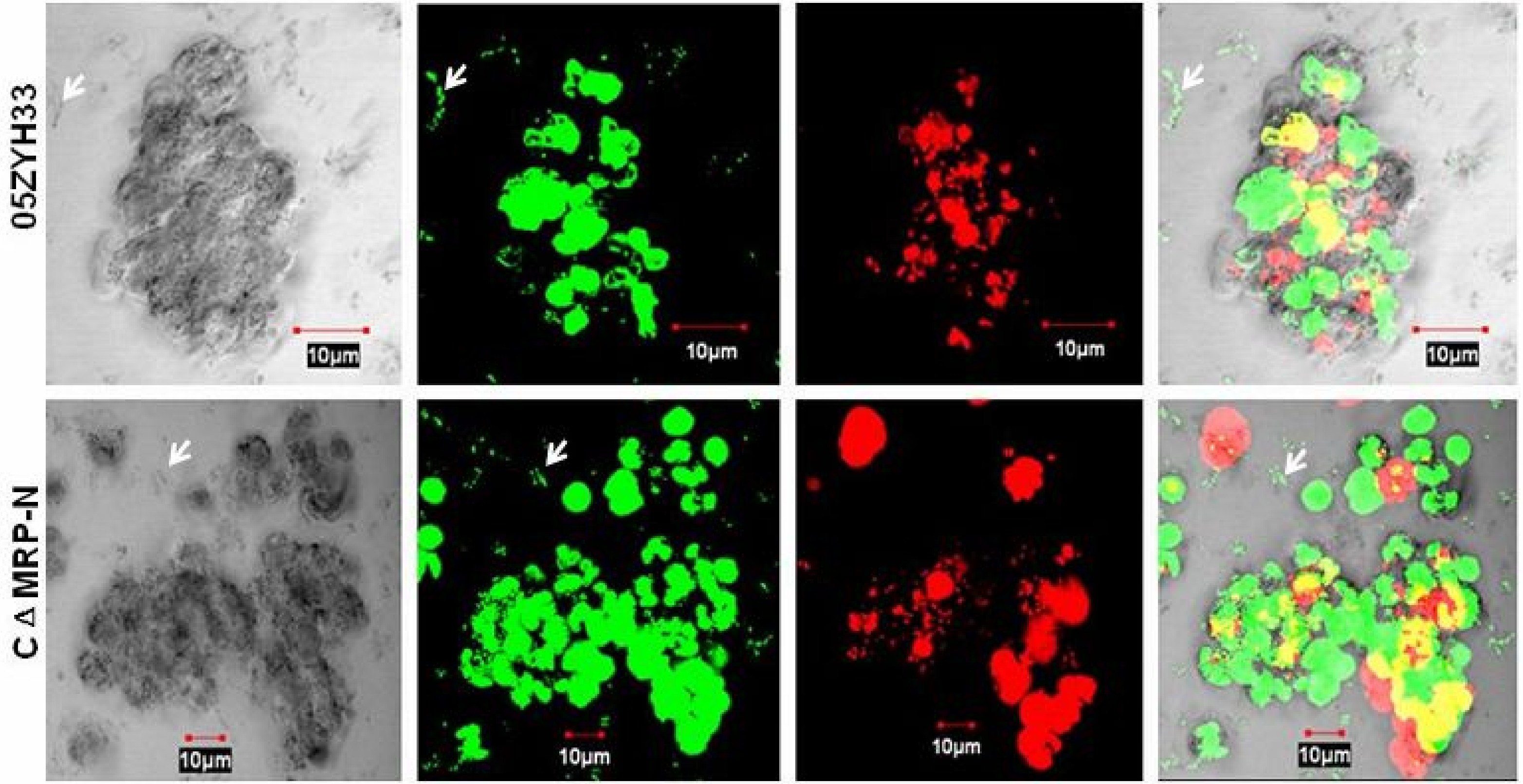

Supplementary Figure 2. Images are enlarged from Figure 6. PMNs incubated with *S. suis* at a multiplicity of infection of 1:15 in 50% serum supplemented with hFg were analysed by confocal immunofluorescence microscopy in the PMN killing assay. Dead and viable PMNs/bacteria were stained by PI (red) and SYTO 9 (green), respectively. The extracellular viable bacteria are indicated by a white arrow. Bar is as indicated in figure.
